# The Role of Cardiac Magnetic Resonance Imaging in the Evaluation of Arrhythmogenic Right Ventricular Dysplasia

**Published:** 2010-12-26

**Authors:** Boban Thomas, Nuno Jalles Tavares

**Affiliations:** Caselas MR Center, Lisbon, Portugal

**Keywords:** magnetic resonance imaging, arrhythmogenic right ventricular dysplasia

Arrhythmogenic right ventricular cardiomyopathy/dysplasia (ARVC/D) is a cardiac muscle disorder characterized pathologically by fibrofatty replacement of the right ventricular (RV) myocardium [1]. In the early stage of the disease, structural changes may be confined to, the so called "triangle of dysplasia" in the RV. Clinical expression ranges from an asymptomatic phenotype, arrhythmias or even ventricular systolic dysfunction (right or left or both). Although the word 'right' has remained prominent in the description of the disease, left ventricular involvement is now acknowledged at a stage earlier than conventionally acknowledged [2].

ARVC/D is now recognized to be a genetic disease affecting the desmosomes that are responsible for cell-to-cell binding with mutations eventually affecting gap junction functioning [2]. Other mutations in non-desmosomal genes have been described as well. The link between ARVC/D and myocarditis is controversial and unclear despite recent reports that indicate a similar clinical presentation [3,4].

The original 1994 International Task Force criteria (ITFC) for the clinical diagnosis of ARVC/D focused primarily on RV disease manifestations and mandated the absence of or only mild LV involvement to prevent confounding with commoner diseases such as ischemic heart disease and dilated cardiomyopathy, that may also have an arrhythmogenic substrate.

The 1994 ITFC were derived from clinical experience gained from cases with extreme phenotypic manifestations and survivors of SCD and represented what we know today to be the extreme end of the spectrum of a disease [5]. As a result of this bias, criteria were highly specific (identified the unaffected well because of the need to satisfy rigorous criteria), but they lacked sensitivity for early and familial disease (where the manifestations are less remarkable).

The 1994 ITFC mentioned morphological changes in the RV including alteration in systolic function, dilatation and aneurysmal changes as major criteria if these were severe in nature and relegated them to a minor status if less accentuated. Tissue characterization of the wall to demonstrate fibrofatty replacement of the myocardium on endomyocardial biopsy was also indicated as a major criterion. In the morphological evaluation of the RV, no mention was specifically made about the imaging modality to be used and cardiac MRI was in its infancy at that stage in the evaluation of ARVC. As it became clear that MRI was preferable to evaluate the RV compared to echocardiography, namely transthoracic echocardiography (TTE) and because of the fact that a high signal on some sequences could indicate fatty tissue, MRI began to be adopted for the evaluation of ARVC at a rapid rate despite the unresolved status of the technique in the evaluation of the disease. Some referral centers accumulated large cohorts of patients, studying them rigorously and applying CMR also in the evaluation of these patients [6]. As these centers published their experience and images often from patients with advanced stages of the disease, numerous other centres with MRI units began evaluating patients without fully understanding many of the caveats related to the technique itself. The border between what was normal and clearly abnormal was rather blurred in many situations.

The rather dramatic presentation of SCD or malignant ventricular arrhythmias that may accompany the disease, especially in young individuals, was of most concern to many clinicians and the threshold for clinical suspicion was therefore lowered. The fear of the consequences of a missed diagnosis, also medicolegal, led many clinicians to order a cardiac MRI, considered by some to be a definitive test while often ignoring to perform many other indicated tests to fulfil ITFC. The ability to assess for major criteria and minor criteria made MRI a promising technique despite the subjectivity inherent in the interpretation of wall thinning, wall motion, and intramyocardial fat. Despite lacking standardized CMR protocols and studies systematically comparing it with other imaging techniques, a problematic practice pattern emerged which was highlighted in a report from the highly experienced group at Johns Hopkins on the misdiagnosis of ARVC/D [7] (Figure 1,2). A few salient findings of this landmark study merit emphasis. 92% of the patients who underwent a MRI study previously in centers around the US prior to the reevaluation at the Hopkins center were reported to have an abnormal study. In 76% of these patients (the ones with an abnormal study), the only findings were intramyocardial fat/wall thinning. None of these findings were confirmed on re-evaluation and none of these patients eventually were diagnosed with ARVC based on the 1994 ITFC. Remarkably on re-evaluation, only 27% of the 89 patients were confirmed to have ARVC whereas all had been advised to undergo placement of an ICD and 34% had already done so. The ECG and a TTE were the only exams that were performed more frequently than a cardiac MRI.

The purpose of this review is to critically evaluate the role of MRI in ARVC and to emphasize the redefined role of MRI as proposed in the revision of the original ITFC, which was published recently [2]. In an era of surfeit of information, the authors believe that numerous comprehensive reviews about ARVC exist in the literature and a few specific ones regarding the role of CMR [8]. This is not meant to be a complete review of ARVC but one that provides some practical advice for a practitioner (often in nonreferral centers) who encounters a probable clinical diagnosis of ARVC and is considering the use of CMR to evaluate the patient as part of a comprehensive work-up.

## What is the added value that CMR adds in the evaluation of ARVC?

**Figure 3:**
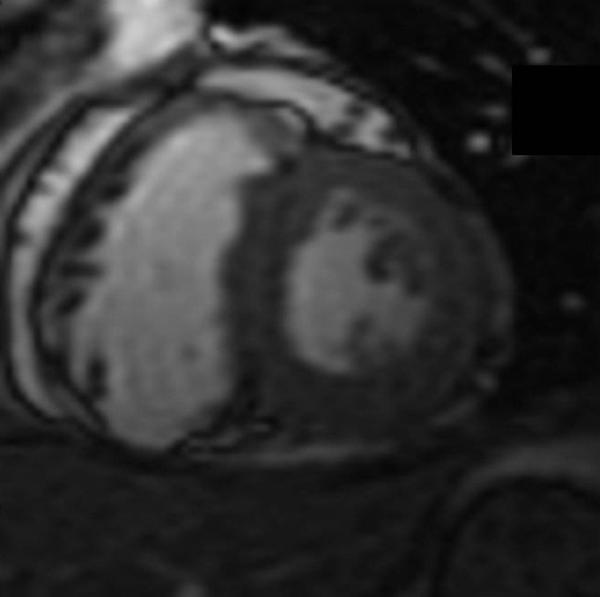
The inferior RV wall shows obvious bulging in a patient who was diagnosed with ARVC after satisfying ITFC.

**Figure 4:**
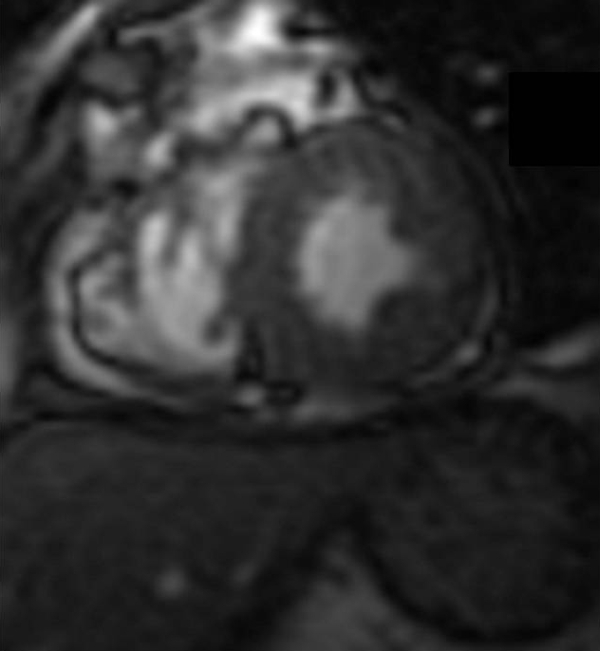
The RV free wall shows various areas of aneurysmatic changes in the same patient in the short-axis view.

**Figure 5:**
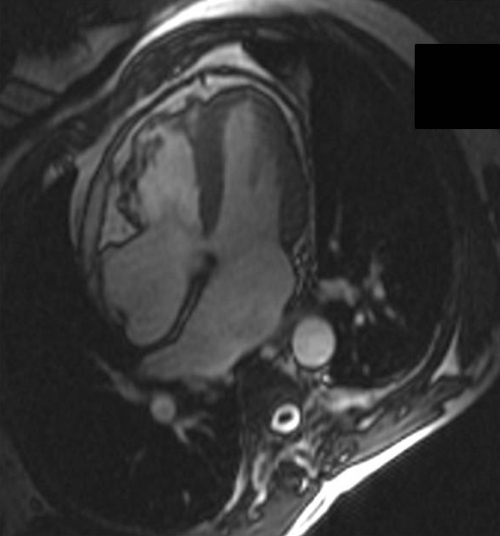
The aneurysmatic changes are more obvious on the four-chamber view.

Morphological and functional evaluation of the RV is greatly facilitated by currently available MR techniques. Cine sequences help in the assessment of wall motion abnormalities if present and in the volumetric quantification of RV function. A major departure from the 1994 ITFC in the revision published recently has been the provision of specific numbers for both ejection fraction and end-diastolic volume indexed to body surface area [2]. The current criteria require the presence of a WMA (Figure 3,4,5) and an increased RVEDV or decreased EF for one major or minor criterion to be fulfilled (distinguished by the severity of the quantitative parameters). A qualitative parameter (WMA) and one quantitative (either EF or indexed EDV) is therefore required together.

The current revision therefore clearly defines the following MRI parameters to be considered to fulfil a major or minor criteria:

**MAJOR:** Regional RV akinesia or dyskinesia or dyssynchronous RV contraction ***and*** 1 of the following:

 - Ratio of RV end-diastolic volume to BSA ≤110 mL/m^2^ (male) or ≤100 mL/m^2^ (female)

- ***or*** RV ejection fraction ≤40%

**MINOR:** Regional RV akinesia or dyskinesia or dyssynchronous RV contraction ***and*** 1 of the following:

- Ratio of RV end-diastolic volume to BSA ≤100 to <110 mL/m^2^  (male) or ≤90 to <100 mL/m^2^ (female)

- ***or*** RV ejection fraction >40% to ≤45%

There is no mention of fatty infiltration, trabercular disarray, wall thinning or delayed enhancement suggestive of fibrosis and current data are incomplete and rather preliminary to consider mentioning LV involvement but subsequent revisions, which hopefully will be more regular may take note of this changed paradigm.

Because ARVC is a progressive disease that spans the spectrum from being completely asymptomatic to presenting as SCD, morphological abnormalities begin to appear and worsen over time and it is unclear which is the first abnormality to appear - e.g ECG vs CMR vs SAECG vs Holter. It is intuitive that as phenotypic manifestations appear abnormalities on various tests appear either sequentially or concomitantly. It is our experience and that of others [8] that it is unlikely to have an abnormal CMR scan with other tests reported as normal. Therefore in the frankly manifested cases CMR and other tests will show abnormalities and a diagnosis may sometimes be clinched without a CMR study. This was confirmed recently in the the Multidisciplinary study of Right Ventricular Cardiomyopathy/Dysplasia which established the North American ARVC/D Registry [9].

**Figure 6:**
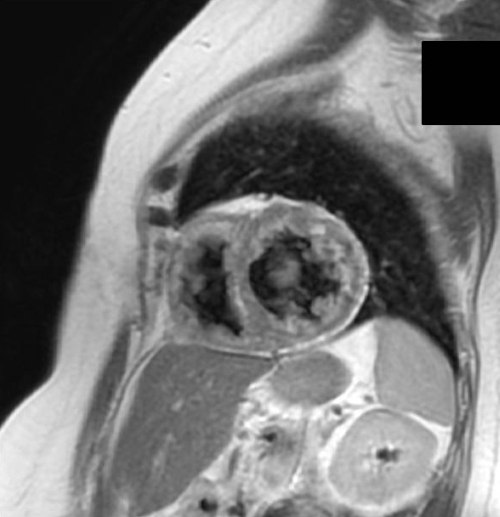
The normal LV has a grayish appearance on this sequence often labeled a black-blood sequence (the blood filled cavities have no signal) and fat gives off a high signal appearing white (the subcutaneous fat has a very high signal). In this patient the grayish LV wall is interspersed in various areas with high signal most likely to be fat in this patient who presented with ventricular tachycardia.

**Figure 7:**
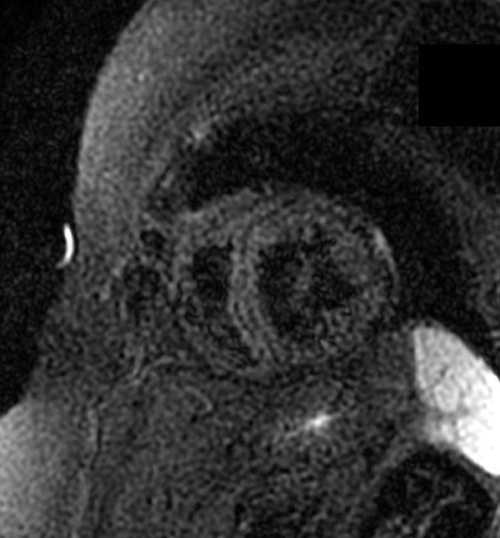
Using a sequence that suppresses the high fat signal, the white patches in the LV (also noted as a linear streak in the septum) in figure 6 are confirmed to be fat. There is also no signal from the subcutaneous fat. This patient was found to have a desmoplakin mutation.

There are some situations where a CMR study provides additional information that may be unique. Although ARVC has traditionally been designated a disorder affecting the RV and the original criteria specified the absence of LV involvement, recently this paradigm has been challenged by the publication of a report of patients with predominantly mutations in the desmoplakin gene and marked LV involvement with relative sparing of the RV [10]. The group emphasized the differentiation from dilated cardiomyopathy based on a clinical presentation dominated by arrhythmias and fewer symptoms suggestive of congestive heart failure. Moreover a very specific type of delayed enhancement was seen after administration of gadolinium which involved the midmyocardial or subepicardial regions. There are changes in the ECG which may suggest this variant of ARVC designated as left-dominant arrhythmogenic cardiomyopathy. Another situation where CMR may provide additional value may be in the screening of relatives of patients with diagnosed ARVC. The Johns Hopkins group carefully selected asymptomatic relatives of patients from their registry and were able to demonstrate that carriers of mutations in some genes (mainly plakophilin - 2) were likely to have a very specific WMA in the subtricuspid region that they designated the ***accordion sign*** attributable to a crinkling effect that accentuated in systole [11].  However, they could not find prominent LV involvement in PKP2 in their cohort unlike the group that eventually described the LDAC variant. Remarkably all four of the patients with a mutation for DSP demonstrated fatty infiltration of the LV (Figure 6,7).  It is possible that LV involvement may be more prominent or confined to those with a mutation in the DSP gene and the accordion sign may be more common among carriers with the PKP2 mutation. Targeting asymptomatic family members of index cases with mutations in either DSP or PKP2 for screening using CMR may therefore be a reasonable strategy. It must be emphasized that the study which demonstrated the utility of the accordion sign included subjects who were truly asymptomatic and did not seek medical attention but were family members of probands who were offered genotyping and a CMR study. 

Although the RVOT is well evaluated by CMR, and even though one group has suggested early involvement of the RVOT as a forme-fruste of ARVC, in some cases labelled clinically as idiopathic RVOT VT [12], recent work has suggested that the RVOT involvement and dilatation seems to accompany generalized RV involvement especially when investigated by CMR [9,11]. Therefore currently available CMR data indicate concurrent dilatation and involvement of the RVOT and the main body of the RV. Isolated RVOT involvement is not detected by CMR.

## What is the role of CMR in patients with an initial clinical presentation suspicious for ARVC?

Most robust studies have come out of referral centers including patients with established disease according to the 1994 TFC and CMR studies were usually performed in these probands and often offered to family members if they met what was designated as modified TF criteria for familial ARVC [6,10,11,13]. The inclusion of a mixture of patients who fulfilled original or 'modified' TF criteria for familial disease where a family member with 200 ventricular ectopics could qualify for a diagnosis of ARVD/C may be rather liberal as it does not exclude the possibility of inclusion of patients with other cardiomyopathies [10,13] (the possibility of a family member having another cardiomyopathy is not totally excluded obviously in such circumstances).

As patients with ARVC may present at virtually any clinical practice it is important to properly identify these patients and do a comprehensive work up. These de novo patients have often presented some of the most difficult situations and misdiagnosis was not an unusual phenomenon as previously reported. One culprit in misdiagnosis was the inaccurate interpretation of CMR studies, the overuse of CMR compared to other diagnostic modalities and even ignorance regarding the TFC [7]. The Multidisciplinary study of Right Ventricular Cardiomyopathy/Dysplasia established the North American ARVC/D Registry (North American Multidisciplinary study, NAMS) which consisted of 18 enrolling centers to address this problem partially because some of these centers were experienced referral centers and may not represent general cardiology practices where patients may present with symptoms suggestive of ARVC/D [9]. The fact that only three centers enrolled more than ten patients underscores the relative rarity of the disease. The commonest symptoms were palpitations, syncope, dizziness or chest pain and 38 had sustained clinical VT.The role of MRI in the newly defined criteria in 2010 was determined from the study [2]. To exclude a bias in the determination of the sensitivity and specificity of any test in the diagnosis of ARVC, proband data were excluded if that test was crucial for the diagnosis of the individual patient because while establishing diagnostic criteria and in determining the sensitivity and specificity of a new screening test, it is recommended that the primary diagnosis should have been arrived at without the particular test being evaluated. Compared to the MRI studies of 462 normal subjects there was a clear decrease in RVEDV indexed to BSA and RVEF in both males and female probands (n= 44). This was the basis for the inclusion of volumetric criteria by MRI in the Revised TFC of 2010. Post hoc application of this Revised version showed an increase in sensitivity compared to the original ITFC. A panoply of tests are often used in these patients ranging from echocardiography, electrocardiography, angiography, SAECG, Holter, RV biopsy and MRI. A sequential analysis revealed that evaluating the RV echocardiogram, RV angiogram, SAECG complemented by the ECG and the Holter provided the best diagnostic approach even excluding MRI and biopsy. Indeed when a 7-variable model (which included all the tests applied) was compared to a best 6-variable model (where one test was excluded one and a time and the effect of this approach on diagnostic capability re-evaluated), the exclusion of MRI seemed to cause the least effect on the diagnostic capability of the 6-variable model [9]. This contradicted the general practice pattern that revealed a misdiagnosis of ARVC with overreliance on MRI [9]. However, MRI is noninvasive compared to a biopsy and the angiogram and therefore has a clear role in the evaluation of these patients.

## What is the relationship between myocarditis and ARVC?

There is one school of thought that suggests that acute episodes of inflammation or myocarditis may be a stage in the evolution of ARVC [3]. Both myocarditis and ARVC can be studied by CMR and recently a very specific set of criteria for myocarditis with a dominant role for MRI was described [14]. One group has even suggested that myocarditis may even mimic ARVC upto the point of even fully satisfying the original ITFC [3]. However, the findings of this study were hotly contested [4]. Clinical difficulties may arise if a patient presents with symptoms and signs suggestive of myocarditis but there is a family history of ARVC. The morphological abnormalities detected on CMR should be reported whether they suggest myocarditis or ARVC. Delayed enhancement may be present in both and some patterns may be similar (the subepicardial involvement in both) when they involve the LV. What is important is that the diagnosis of ARVC should be made by the clinical cardiologist or the electrophysiologist who makes the therapeutic decisions and not by the physician who interprets the CMR study. At this point the controversy regarding the link between the two entities has not been settled.

## Practical difficulties in studying patients with ARVC

It must be emphasized that the evaluation and detection of fat or signal suggestive of fat is completely excluded from the MRI evaluation of ARVC. While advanced cases of ARVC may show extensive fat deposition, early stage disease even with microscopic infiltration cannot be detected by MRI based on a high signal [15,16]. A high signal on some sequences should not be assumed to be fat except in the most extraordinary situations (for e.g. in the same region where there is a WMA). Numerous technical factors elucidated by others have emphasized why a high signal in the region of the RV is not unusual and it may be difficult to clearly separate the epicardial fat from the myocardium. What confounds it further is that in ARVC fibrofatty infiltration commences from the epicardium and is directed inwards. Moreover fat can be found in the myocardium of patients without ARVC. It is therefore appropriate that the search for fatty infiltration is not recommended. Even experienced groups have demonstrated that although qualitative assessment of RV structure and function is highly reproducible, fat infiltration is less reproducible and lacked specificity compared with RV kinetic abnormalities [16].

Standard protocols have been published but each center will have to optimize their protocols to include one state-of-the art cine sequence which will usually be a steady state free precession sequence (SSFP) with a conventional gradient echo type sequence as a back-up in case of the SSFP sequence failing in some patients with frequents ectopics. The optimization of a cine sequence is fundamental because volumetric and kinetic analysis forms the backbone of a CMR study for ARVC/D. However, contouring RV volumes is a more difficult task compared to the LV as the difficulty is most obvious in the most basal slices where the atrioventricular junction moving in and out of the imaging plane can cause some confusion. There are also the slices where the RVOT ending in the pulmonary valve have to be contoured meticulously. The propensity for a bias to over or underestimate the volumes can occur if the history is known. While volume analysis is inherently more objective, WMA can be more challenging. The RV has a complex shape and movement and based on the internal trabecular structure a subject can have some untethered regions that lag behind and may look dyskinetic. To the inexperienced some of these areas may look aneurysmal. It is not only the inexperienced that may have some difficulty in interpreting WMA or just wall motion. Even experienced observers may be deceived into classifying some movements in an unaffected RV as abnormal [17]. While the emphasis on the need to also document quantitative dysfunction (increased RVEDV indexed to BSA or decreased EF) may provide some cushion from errors, this is no guarantee. According to the current modification ***a patient with quantitative dysfunction (even if both RVEDV and EF) will not satisfy one parameter in the Revised TFC if there are no WMA detected***. 

A tomographic black blood sequence is also helpful to elucidate fat in the LV in those with DSP mutations. A sequence to study fibrosis after gadolinium using the standard delayed enhancement technique [18] is included in all cardiac packets sold by vendors and optimization has been made easier by software modifications that help select the optimal inversion time to null the myocardium. In practice we have not found any exceptional advantage to this modification which is sometimes offered at considerable additional cost and a good understanding of the architecture of the sequence is all that is required to acquire excellent images. Currently vendors offer these sequences in pre-packaged formats for most machines often excluding the capability to manipulate sequence parameters in some cases, which was possible previously. Although it is intuitive that most CMR practitioners need to have a reasonable understanding of the physics and the architectural composition of the common sequences, it is not always the case. For optimal image acquisition it is important to be able to manipulate some sequence parameters to adapt to each subject. As cine sequences in CMR are currently not real-time but an average of approximately 8-16 cardiac cycles in most cases, the occurrence of ectopics during some loops as the images are acquired from the base to the apex may cause some of the loops to be out of phase with the others. While contouring for ventricular volumes it should be confirmed that the right phases are added together to calculate systolic and diastolic volumes. Some commercial software may have some limitations when this happens and a tedious manual approach may have to be resorted to. In the sequence to elucidate any potential delayed enhancement after gadolinium, it is important to realize that a high signal may mean either fat or fibrosis [18] and a black blood sequence optimized for fat detection should be done to clarify this. Although a lot has been written about delayed enhancement in the RV, at this moment it is still not as reliable in comparison to detection of a high signal in the LV due to various technical reasons. What is important is that high signal from epicardial fat should not be confounded with fibrofatty replacement of the RV free wall.

In the interpretation of studies the presence of mimics of ARVC/D should be considered. Sarcoidosis is an oft-quoted mimic demonstrating WMA very similar to ARVC/D. Structural changes in the RV have also been reported recently in patients with the Brugada syndrome [19]. A complete or partial absence of the pericardium can give images that may resemble ARVC. These examples demonstrate that CMR is not a stand-alone technique in the evaluation of ARVC/D and certainly not a gold-standard.

Despite these difficulties the results of a study from the Brompton group revealed that intraobserver concordance was very high and fidelity of readings was maintained even after a year in a blinded analysis [20]. High interobserver concordance was noted with a reader who progressively gained experience alongside a more established reader and the lowest concordance was confirmed for the least experienced reader. This indicated that CMR patterns, especially WMA in ARVC can be learned over a period of time.

For those patients with numerous extrasystoles ECG gating may be problematic with some image degradation but hardware and software improvements have mitigated some of these problems. In some patients with frequent extrasystoles, a beta blocker prior to the study may help. Finally for all practical purposes, patients with ICDs implanted should not undergo a CMR study as there are other alternatives and although it may be done in highly experienced centers, it may be prudent to avoid CMR studies in these patients.

## Conclusion

Cardiac MRI is a valuable imaging modality in the evaluation of patients with ARVC/D. It can be used currently to evaluate for criteria specific to MR evaluation of these patients as stated in the Revised TFC published recently in order to reach a diagnosis [2]. It may also provide morphological information of some variants of the disease that may initially and primarily involve the left ventricle [10]. It may also be used to evaluate asymptomatic family members who may potentially be carriers of some well characterized mutations in components of the desmosome [11]. It may also provide an alternative diagnosis in some cases excluding the disease given some of the unique advantages that MRI offers. Finally a negative CMR study does not exclude ARVC.

## Figures and Tables

**Figure 1:**
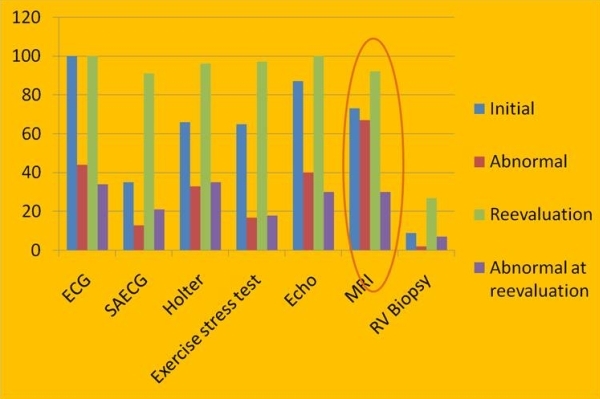
Shows that MRI was one of the most commonly ordered in various centres to rule out ARVC and a larger percentage of MRI studies were considered abnormal in comparison to other tests like signal averaged ECG and RV biopsy. [Reproduced with permission from the publisher John Wiley and Sons. Bomma C, Rutberg J, Tandri H, Nasir K, Roguin A, Tichnell C, Rodriguez R, James C, Kasper E, Spevak P, Bluemke DA, Calkins H: Misdiagnosis of arrhythmogenic right ventricular dysplasia/cardiomyopathy. J Cardiovasc Electrophysiol 2004 , 15(3):300-306].

**Figure 2:**
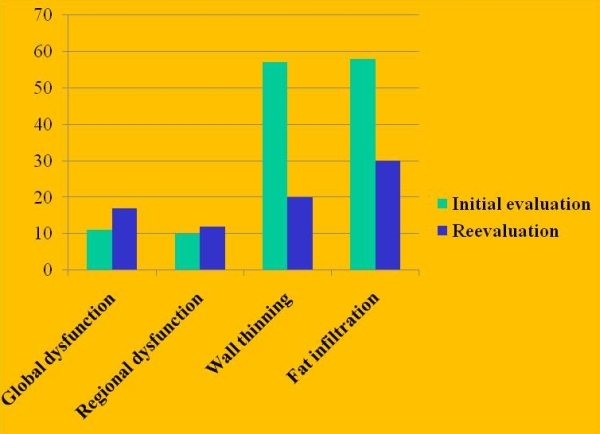
In the initial evaluation a large number of studies reported wall thinning and fat infiltration on the CMR study that was not subsequently confirmed at the more experienced referral centre where these subjects sought a second opinion. [Adapted from Bomma C, Rutberg J, Tandri H, Nasir K, Roguin A, Tichnell C, Rodriguez R, James C, Kasper E, Spevak P, Bluemke DA, Calkins H: Misdiagnosis of arrhythmogenic right ventricular dysplasia/cardiomyopathy. J Cardiovasc Electrophysiol 2004 , 15(3):300-306].
